# Determining the Cause of Coronary Vasomotor Disorders in Patients With Ischemia and Nonobstructive Coronary Arteries: Design and Rationale of the DISCOVER INOCA Prospective, Multicenter Registry

**DOI:** 10.1016/j.jscai.2024.102046

**Published:** 2024-05-03

**Authors:** Samit M. Shah, Jennifer A. Tremmel, Timothy D. Henry, Nathaniel R. Smilowitz, Megha Prasad, Yuhei Kobayashi, Glen A. Henry, Habib Samady, Bruce A. Samuels, Amir Lerman, Jeffrey W. Moses, Cody Pietras, Zhiyuan Zhang, Daniela Tirziu, Helen Parise, Ecaterina Cristea, Daniel Chamié, Daniel Grubman, Kyna Henrici, Elzar Matmusaeva, Nida Latif, Natasha Cigarroa, Alexandra J. Lansky

**Affiliations:** a Section of Cardiovascular Medicine, Department of Internal Medicine, Yale School of Medicine, New Haven, Connecticut; b Veterans Affairs Connecticut Healthcare System, West Haven, Connecticut; c Yale Cardiovascular Research Group, Yale School of Medicine, New Haven, Connecticut; d Department of Medicine, Division of Cardiovascular Medicine, Stanford University School of Medicine, Stanford, California; e The Christ Hospital Heart and Vascular Institute, Cincinnati, Ohio; f The Carl and Edyth Lindner Research Center at The Christ Hospital, Cincinnati, Ohio; g Leon H. Charney Division of Cardiology, Department of Medicine, NYU Grossman School of Medicine, New York, New York; h Cardiology Section, Department of Medicine, VA New York Harbor Healthcare System, New York, New York; i Department of Medicine, Division of Cardiology, Columbia University Irving Medical Center, New York, New York; j NewYork-Presbyterian Brooklyn Methodist Hospital/Weill Cornell Medical College, New York, New York; k Northeast Georgia Medical Center, Gainesville, Georgia; l Smidt Heart Institute, Cedars-Sinai Medical Center, Los Angeles, California; m Department of Cardiovascular Medicine, Mayo Clinic, Rochester, Minnesota; n St Francis Hospital & Heart Center, Roslyn, New York; o Department of Internal Medicine, Yale School of Medicine, New Haven, Connecticut

**Keywords:** coronary, intravascular imaging, physiology, stable ischemic heart disease

## Abstract

**Background:**

Approximately 30% to 50% of patients who are referred for diagnostic coronary angiography are found to have no obstructive coronary artery disease (CAD). Ischemia and nonobstructive coronary arteries (INOCA) is increasingly recognized and encompasses coronary microvascular dysfunction, vasospastic angina, symptomatic myocardial bridging, and other vasomotor disorders. However, the prevalence of these disorders and whether underlying atherosclerotic plaque burden and morphology affect the long-term outcomes of each physiologic phenotype is unknown.

**Methods:**

The DISCOVER INOCA registry is ongoing at 8 centers in the United States and plans to enroll 500 patients with ischemic heart disease referred for angiography undergoing coronary function testing (CFT). All participants will complete patient-reported outcome measures and undergo protocol-guided angiography, acetylcholine provocation, coronary thermodilution, and intravascular imaging. Follow-up assessments occur at 30 days, 6 months, 1 year, and annually for 5 years. The primary short-term end point is the prevalence of INOCA phenotypes based on physiology and the degree of atherosclerosis based on intravascular ultrasound or optical coherence tomography (intravascular imaging). The primary long-term end point is the incidence of major adverse cardiovascular events, defined as a composite of cardiovascular death, myocardial infarction, hospitalization for cardiovascular causes, or coronary revascularization at a follow-up of 5 years. At the time of this publication, 100 participants have been enrolled.

**Conclusions:**

DISCOVER INOCA is the first prospective study of INOCA patients to integrate anatomic and physiologic measures of disease and correlate them with long-term outcomes. DISCOVER INOCA will report on the prevalence of INOCA phenotypes, the safety of comprehensive invasive CFT, and the impact of testing on diagnoses and medical therapy. Symptoms and cardiovascular adverse events at long-term follow-up will be determined in patients with no obstructive CAD undergoing angiography.

## Introduction

Among patients referred for invasive coronary angiography (ICA) with or without ischemia on noninvasive stress testing, nearly 50% are found to have no obstructive coronary artery disease (CAD).^1^, ^2^, ^3^ Myocardial ischemia and nonobstructive coronary arteries (INOCA) is an umbrella term encompassing specific syndromes including coronary microvascular dysfunction (CMD) due to structural abnormalities or abnormally elevated resting flow, vasospastic angina (VSA), diffuse physiologically significant epicardial CAD, endothelial dysfunction, and symptomatic myocardial bridging. Previous studies suggest that there are 3 to 4 million people in the United States who suffer from INOCA.^4^^,^^5^ Compared with patients with obstructive CAD, these patients are more likely to suffer from anxiety, depression, and recurrent symptoms.^6^ Furthermore, compared with healthy controls, these patients are at increased risk for major adverse cardiovascular events (MACE) and cardiovascular mortality.^7^^,^^8^

Invasive anatomic and physiologic assessment of patients with INOCA has revealed epicardial endothelial dysfunction, CMD, myocardial bridging, or diffuse epicardial atherosclerosis in >75% of patients.^2^ Recent studies have shown that angina and quality of life can be improved when coronary physiology assessment is incorporated into clinical care.^9^^,^^10^ However, comprehensive assessments of coronary physiology (including the epicardial vessels and microvascular bed) with coronary function testing (CFT) are currently not performed routinely in most medical centers despite being recommended by professional society guidelines.^6^^,^^11^^,^^12^ The rationale for performing intravascular imaging and comprehensive coronary physiology assessment in patients with INOCA is that ICA alone cannot diagnose or exclude CMD, diffuse atherosclerosis, physiologically significant myocardial bridging, endothelial dysfunction, or dynamic processes such as coronary vasospasm.^9^ Additionally, determining the specific INOCA physiologic phenotype can help guide medical therapy and long-term prognosis.^6^^,^^13^

This prospective, multicenter registry will enroll up to 500 patients presenting with symptomatic ischemic heart disease and referred for ICA based on clinical indications. The overall objective of the Determining the Cause of Coronary Vasomotor Disorders in Patients With Ischemia and Nonobstructive Coronary Arteries (DISCOVER INOCA) Registry is to characterize phenotypes of INOCA and long-term outcomes based on both an anatomic evaluation (ICA and intravascular imaging) and physiologic assessment with the CoroFlow Cardiovascular System (Abbott Vascular) (Central Illustration). In this study, we aim to: (1) describe the prevalence of the INOCA phenotypes: CMD, VSA, mixed CMD/VSA, myocardial bridging, and other disorders of coronary physiology; (2) characterize the burden of epicardial coronary artery atherosclerosis and myocardial bridging; and (3) characterize the natural history and outcomes of patients with INOCA and determine variables associated with MACE.

**Central Illustration:**
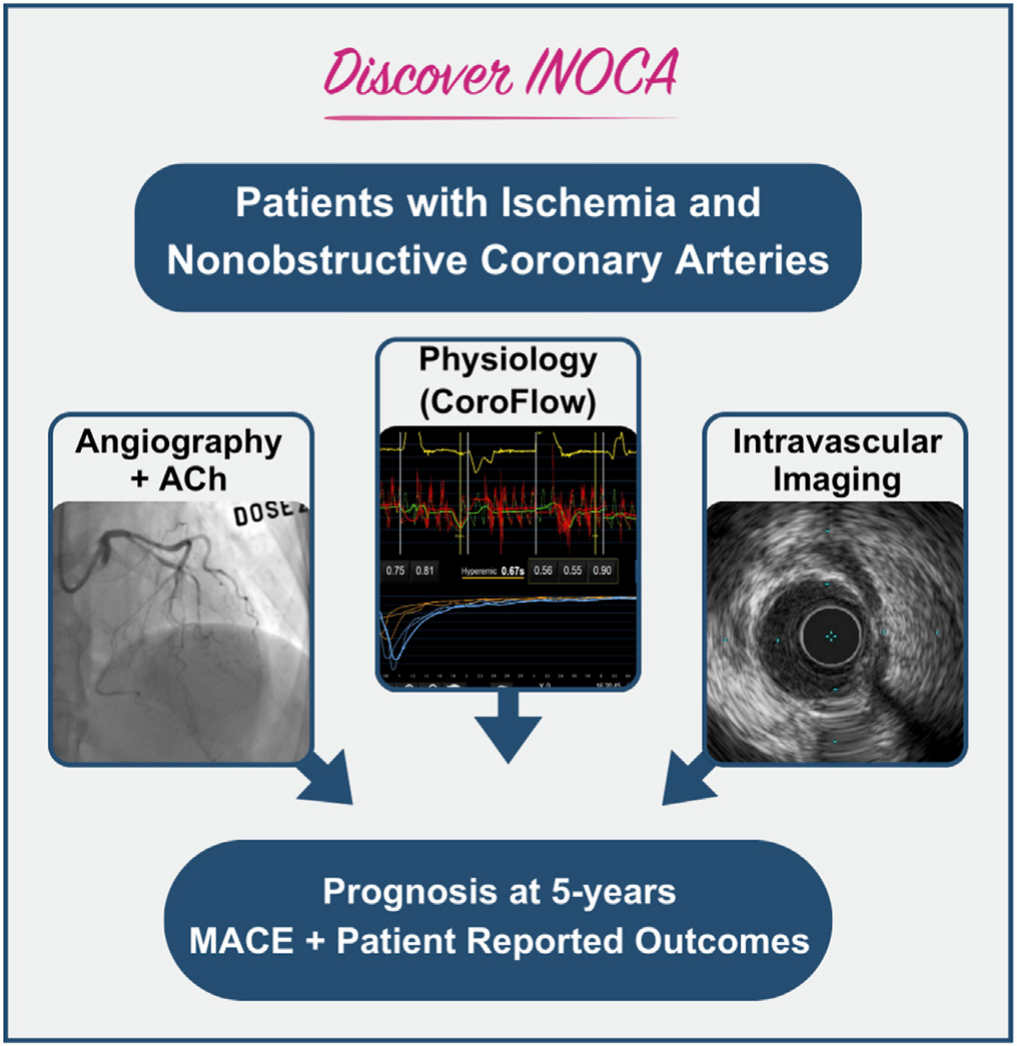
The DISCOVER INOCA multicenter registry aims to identify specific phenotypes of ischemia and nonobstructive coronary arteries through combined anatomic and physiological assessments (CoroFlow Cardiovascular System) and determine long-term outcomes. MACE, major adverse cardiovascular events (composite of cardiovascular death, myocardial infarction, hospitalization for cardiovascular causes, or coronary revascularization).

## Methods

### Study design

DISCOVER INOCA is a prospective, multicenter registry of patients with INOCA evaluated by ICA, intravascular imaging, and physiologic assessment with the CoroFlow Cardiovascular System (Central Illustration). The study is registered at ClinicalTrials.gov (NCT05288361). Participants will be enrolled at up to 10 investigational sites in the United States with prior experience performing CFT. A current list of investigational sites is provided in Supplemental Table S1. Clinical end points will be adjudicated by independent physician review by a provider who is not part of the study team. The relationship of these events to the procedure and physiology assessment protocol will also be adjudicated. Index procedure angiographic data, physiologic assessments, and intracoronary imaging will be analyzed offline by an angiographic core laboratory and intravascular imaging core laboratory (Yale Cardiovascular Research Group).

### Screening process and eligibility criteria

**Figure 1:**
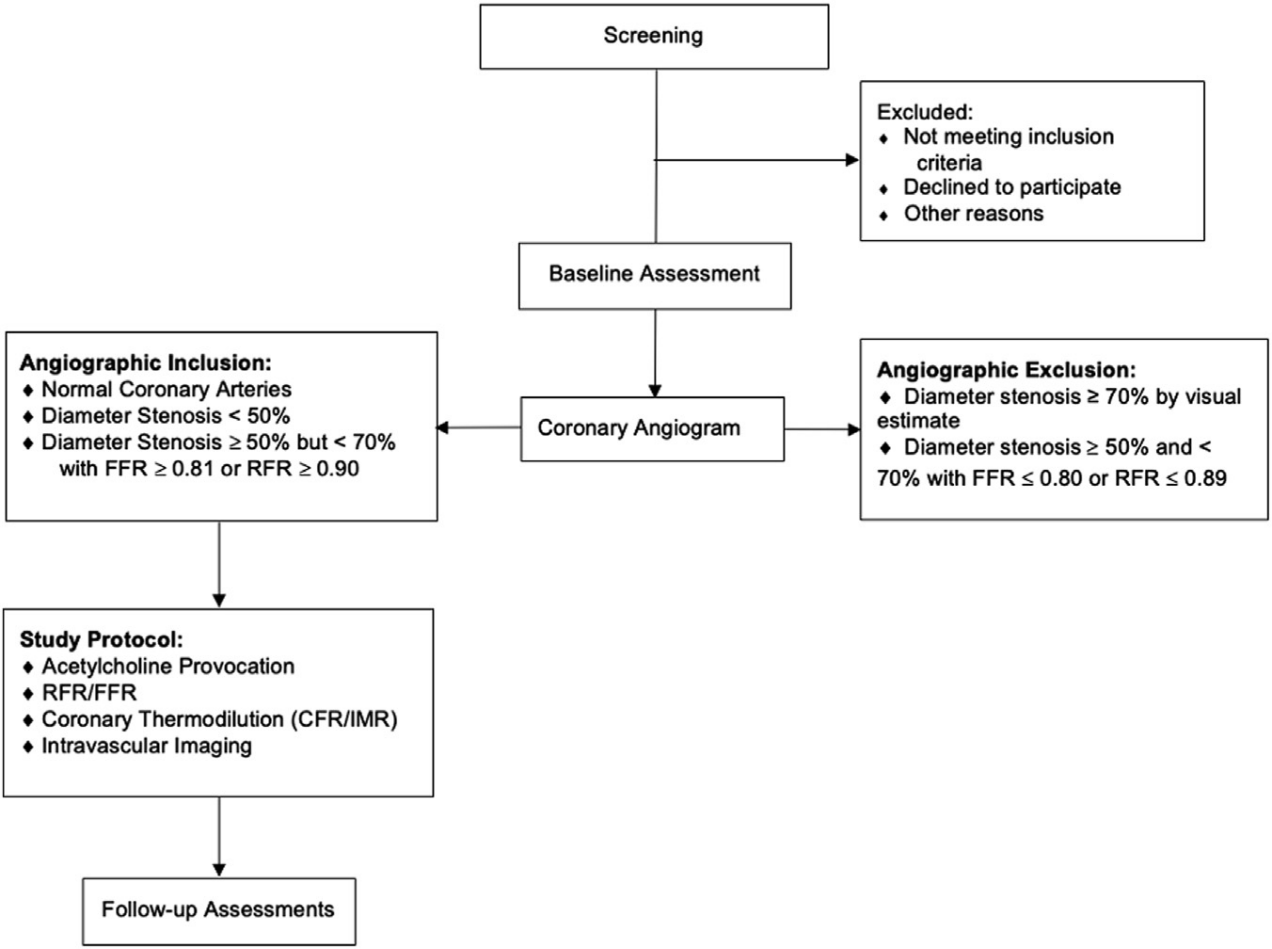
**DISCOVER INOCA registry patient flow diagram.** Patients who meet all inclusion and no exclusion criteria will be eligible for enrollment. CFR, coronary flow reserve; FFR, fractional flow reserve; IMR, index of microcirculatory resistance; RFR, resting wave-free ratio.

Patients with suspected ischemic heart disease that are scheduled for clinically indicated cardiac catheterization at the recommendation of their physician will be screened for inclusion in the registry. Participants must speak English or Spanish as the primary language. Up to 500 eligible patients who meet all inclusion criteria (Table 1) and no exclusion criteria (Table 2) will be enrolled in the study. The point of enrollment is investigator certification at the completion of the index procedure cardiac catheterization that all eligibility criteria are met. Participants will remain enrolled in the study regardless of the findings of the study procedure, including those with normal coronary physiology. The study flow diagram is presented in Figure 1. The following study measures must be assessed prior to the index procedure: Canadian Cardiovascular Society Anginal Classification, Seattle Angina Questionnaire, EuroQol 5 Dimensions – 5 Levels, Patient Health Questionnaire-8, and Generalized Anxiety Disorder-7, as well as preprocedure medications.

**Table 1:** DISCOVER INOCA registry inclusion criteria.

Potential subjects must meet all of the following criteria to be eligible for inclusion in the study:
**General inclusion criteria:** • The patient is a male or nonpregnant female ≥18 y of age • The patient has suspected ischemic heart disease and is referred to undergo clinically indicated invasive coronary angiography • The patient has no obstructive coronary artery disease (CAD) as defined by (1) angiographically normal coronary arteries OR (2) nonobstructive CAD with angiographic stenosis <50%, or ≥50 but <70% with FFR ≥0.81 or RFR ≥0.90 • The patient is willing to comply with specified follow-up evaluations. The patient or legally authorized representative has been informed of the nature of the study, agrees to its provisions, and has been provided written informed consent approved by the appropriate institutional review board or ethics committee

CAD, coronary artery disease; FFR, fractional flow reserve; RFR, resting full cycle ratio.

**Table 2:** DISCOVER INOCA registry exclusion criteria.

Potential subjects will be excluded if any of the following conditions apply:
**General Exclusion Criteria** 1. Pregnant or nursing patients 2. Any myocardial infarction at index presentation or within 90 d prior to enrollment, defined as any electrocardiogram diagnostic for myocardial infarction OR elevation in serum troponin greater than the upper limit of the site-defined reference range 3. Known LVEF <50% or cardiogenic shock requiring pressors or mechanical circulatory assistance (eg, intra-aortic balloon pump, left ventricular assist device, other temporary cardiac support blood pump) 4. Renal insufficiency, defined as estimated glomerular filtration rate (eGFR) <30 mL/min/1.73 m^2^ (by the Modification of Diet in Renal Disease equation) or dialysis at the time of screening 5. Prior percutaneous coronary intervention 6. Planned percutaneous coronary intervention (PCI) 7. Prior coronary artery bypass graft surgery 8. Prior ST-elevation myocardial infarction 9. History of hypertrophic cardiomyopathy 10. History of infiltrative heart disease (eg, cardiac amyloidosis) 11. New York Heart Association Class IV congestive heart failure 12. Severe mitral regurgitation 13. Severe aortic stenosis 14. Severe pulmonary hypertension (mean pulmonary artery pressure ≥35 mm Hg or echocardiographic right ventricular systolic pressure ≥60 mm Hg) 15. Known history of unrepaired or repaired congenital heart disease 16. Past or pending heart transplant, or on the waiting list for organ transplant 17. Known other medical illness or known history of substance abuse that may cause noncompliance with the protocol, or is associated with a life expectancy of less than 1 year 18. Current or planned participation in a study of an investigational therapy
**Angiographic Exclusion Criteria** 1. Angiographic stenosis in any major epicardial vessel ≥70% by visual estimate 2. Angiographic stenosis in any major epicardial vessel ≥50% and <70% by visual estimate with FFR ≤0.80 or RFR ≤0.89

eGFR, estimated glomerular filtration rate; FFR, fractional flow reserve; LVEF, left ventricular ejection fraction; NYHA, New York Heart Association; PCI, percutaneous coronary intervention; RFR, resting full cycle ratio.

### Index procedure

Invasive CFT is intended to be performed as part of the standard of care evaluation for patients who present to the cardiac catheterization laboratory. After excluding the presence of obstructive CAD, the purpose of the procedure is to evaluate coronary vasoreactivity with acetylcholine provocation, physiology with thermodilution, identify myocardial bridging, and describe the burden of atherosclerosis by intravascular imaging (the DISCOVER INOCA index procedure protocol is included as a Supplemental Appendix).

The target vessel for physiologic analysis and intracoronary imaging will be the vessel in which physiologic assessment is clinically indicated or the left anterior descending artery. Coronary angiography will be performed using a standardized acquisition protocol. Angiograms will be acquired in at least 2 projections for each major artery or branch, and the treating physician will record an estimation of stenosis location and severity. The sequence of procedural elements including acetylcholine testing, wire-based testing, and intravascular imaging are at the discretion of the operator and will be recorded.

**Figure 2:**
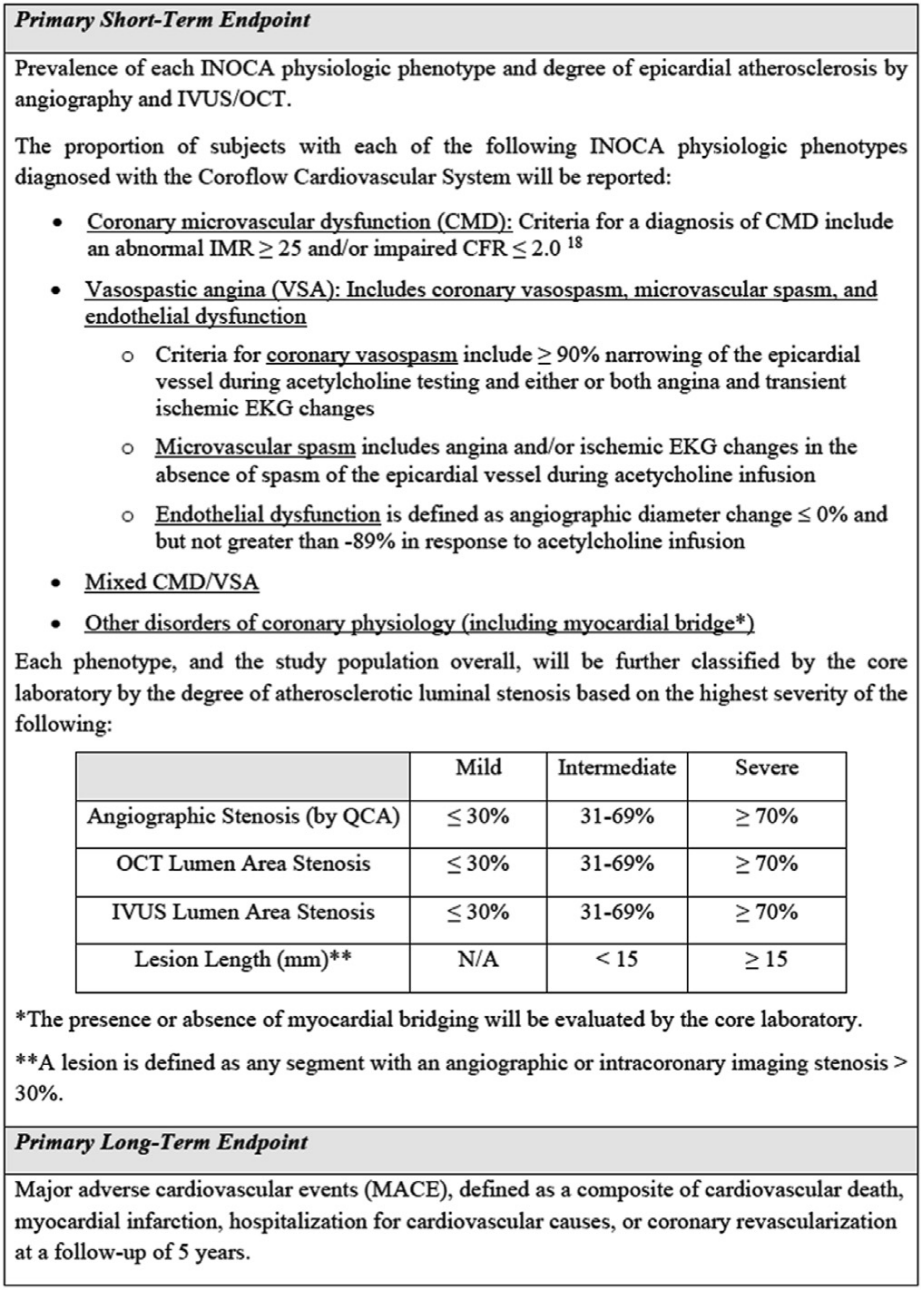
**Primary end points.** Primary short-term and primary long-term end points. CFR, coronary flow reserve, CMD, coronary microvascular dysfunction; EKG, electrocardiogram; IMR, index of microcirculatory resistance; INOCA, ischemia and nonobstructive coronary arteries; IVUS, intravascular ultrasound; N/A, not applicable; OCT, optical coherence tomography; QCA, quantitative coronary angiography; VSA, vasospastic angina.

Coronary vasoreactivity testing will be performed with bolus injection or infusion of escalating doses of intracoronary acetylcholine via a guide catheter. Recommended doses for acetylcholine testing are listed in the Supplemental Appendix. Angiography will be performed after each dose with documentation of electrocardiogram changes and/or symptoms to evaluate for endothelial dysfunction, epicardial coronary vasospasm, and microvascular spasm (Figure 2).

Physiologic assessment will be performed with the CoroFlow Cardiovascular System and PressureWire X guidewire (Abbott Vascular) per the instructions for use. A PressureWire X guidewire will be advanced at least 6 cm or two-thirds of the way down the vessel. Any contrast will be cleared from the catheter, and the temperature will be zeroed. Resting Pd/Pa and resting full cycle ratio will be recorded. Baseline saline thermodilution will be performed and repeated 3 times to determine the baseline mean transit time (T_mn_). Adenosine will be administered intravenously at 140 μg/kg/min. During hyperemia, thermodilution will be repeated 3 times to determine the hyperemic mean transit time (T_mn-hyp_). Coronary flow reserve (CFR), index of microcirculatory resistance (IMR), and fractional flow reserve (FFR) will be recorded (Figure 2).

Intracoronary imaging will be performed with optical coherence tomography (OCT) or intravascular ultrasound (IVUS) according to the acquisition guidelines. Imaging should be performed on the vessel that underwent guidewire assessment using an automated pullback for a minimum of 54 mm.

### Core laboratory analysis

Offline postprocedure analyses will be made by the imaging and physiology core laboratories and include quantitative coronary angiography (QCA) evaluation of all epicardial vessels, quantitative interpretation of IVUS or OCT imaging, resting Pd/Pa, resting full cycle ratio, or other nonhyperemic indices, IMR, CFR, and FFR, and quantitative flow ratio (Medis Medical Imaging).

### Follow-up

All subjects will have clinical follow-up prior to discharge from the index procedure. Changes in medical therapy are at the discretion of the treating provider and will be recorded postprocedure and at all follow-up time points. Screening, procedural, and primary follow-up data collection will occur through the electronic medical record based on the local standard of care, augmented by study-specific telehealth contacts from a centralized call center at 30 days, 6 months, 12 months, and annually through 5 years (Supplemental Table S2). Symptom assessment and patient-reported outcome measures will be completed by the centralized call center at each time point.

### End points

The primary short-term end point is to characterize the prevalence of each INOCA physiologic phenotype and degree of epicardial atherosclerosis (none, mild, moderate) and presence/absence of a myocardial bridge by angiography and IVUS/OCT (Figure 2). The primary long-term end point will be the incidence of MACE, defined as a composite of cardiovascular death, myocardial infarction, hospitalization for cardiovascular causes, or coronary revascularization at a follow-up of 5 years (Figure 2).

#### Secondary end points

Secondary end points are patient-related end points and procedural end points. Secondary patient-related end points include MACE and its components, stroke, progression to obstructive CAD, major bleeding, major vascular complications, angina severity, and patient-reported outcomes (Table 3). Procedural end points include reclassification from preprocedure diagnosis, completion of CFT without device-related serious adverse events or major angiographic complications, and frequency/nature of major angiographic complications (Table 3).

**Table 3:** Secondary end points.

**Patient-Related End Points**
The following end points will be reported at all time points, unless otherwise specified: • MACE at all time points not included in the primary end point • Major adverse cardiovascular and cerebrovascular events (MACCE), defined as MACE and/or any stroke (NeuroARC definition) • All-cause, cardiovascular, and noncardiovascular mortality • Myocardial infarction (Fourth Universal Definition) • Stroke (NeuroARC definition) • Revascularization (PCI or CABG) • Hospitalization for cardiovascular causes • Hospitalization for heart failure • Repeat coronary angiography • Progression to obstructive CAD by core laboratory analysis (subject level) • Progression to obstructive CAD by core laboratory analysis (lesion level) • Major bleeding (BARC definition 3-5) • Major vascular complications • Composite of death, myocardial infarction, and stroke • Angina severity assessed by Canadian Cardiovascular Society class or Braunwald Unstable Angina classification • Seattle Angina Questionnaire as a change from baseline • Quality of life assessed by EQ-5D-5L (at 30 d, 6 mo, and 1-5 y) as a change from baseline • Depression assessed by the PHQ-8 questionnaire (at 30 d, 6 mo, and 1-5 y) as a change from baseline • Anxiety assessed by the GAD-7 questionnaire (at 30 d, 6 mo, and 1-5 y) as a change from baseline
**Procedural End Points**
The following end points will be reported in-hospital (or within 48 h of the index procedure): • Reclassification from preprocedure diagnosis (including noncardiac chest pain) • Completion of the interventional diagnostic procedure without device-related serious adverse events or major angiographic complications • Frequency of major angiographic complications (device-related coronary artery dissection [greater than type C], slow or no flow, or perforation)

BARC, Bleeding Academic Research Consortium; CABG, coronary artery bypass grafting; CAD, coronary artery disease; EQ-5D-5L, EuroQol 5 Dimensions – 5 Levels; GAD, Generalized Anxiety Disorder; MACCE, major adverse cardiovascular and cerebrovascular events; MACE, major adverse cardiovascular events (composite of cardiovascular death, myocardial infarction, hospitalization for cardiovascular causes, or coronary revascularization); NeuroARC, Neurologic Academic Research Consortium; PHQ, Patient Health Questionnaire; PCI, percutaneous coronary intervention.

### Statistical analysis

#### Primary short-term end point

The prevalence of the various phenotypes will be estimated using the Exact (Clopper-Pearson) formula. Assuming the prevalence ranges between 10% and 50% in each phenotype, with a sample size of 500, the width of the CI will range from 5.5% to 8.9% (with a maximum interval width of 8.9%) allowing for adequate estimation of the prevalence within each group.

#### Primary end point

The study sample size was calculated to provide adequate power to identify variables associated with primary long-term end point events on the basis of a range of assumptions about the frequency of high-risk characteristics, their predictive accuracy, the overall rate of such events, and the hazard ratio (HR) for the risk factor. For example, if 32% of patients had a high-risk variable, 500 patients would be needed to provide 70% and 90% power to detect a HR of 4.0 for MACE of 3% and 5%, respectively, with a 1-sided alpha of 0.025. With 500 subjects, the minimum HR that will be able to be detected with event rates of 3% and 5% is 3.0 and 2.3, respectively (assuming 32% prevalence of the high-risk factor as noted above).

Baseline variables that are considered clinically relevant or that show a univariate relationship with outcome will be entered into multivariate Cox proportional hazards regression models. Variables for inclusion will be chosen, given the number of events available, to ensure parsimony of the final models. Lesion-level multivariable models will be adjusted for patient effects by means of the marginal Cox model, and nonsignificant variables dropped by backward selection. Statistical analyses will be performed using SAS software version 9.4 (SAS Institute).

Secondary end points will be reported in the overall population and by phenotype using appropriate descriptive statistics. In general, statistics for continuous variables will include mean, median, quartiles, SD, minimum, maximum, and sample size. Binary variables will be summarized using frequencies, percentages, and sample size. No formal hypothesis testing will be performed, and there is no plan to adjust alpha to account for multiple testing of exploratory secondary end points. Subgroup analyses will be performed for all primary and secondary end points for the following subgroups: presentation, categorization of plaque burden, diabetes status, sex, and plaque characterization.

### Data collection and monitoring

All centers are qualified before participation. During enrollment and follow-up, monitors will review consistency on the electronic data capture system and source documents. Monitoring ensures that the trial complies with current Good Clinical Practice, relevant laws and regulations, and trial protocol, and finally, obtains secure and accurate data.

## Discussion

DISCOVER INOCA is the first prospective, multicenter registry of patients with INOCA undergoing comprehensive CFT with intravascular imaging in the United States. The study is designed to integrate anatomic and physiologic measures of disease and correlate them with long-term outcomes. The standardized CFT protocol was designed by the study executive committee and is shared in its entirety in the Supplemental Appendix. The design of DISCOVER INOCA is unique due to required protocol elements of ICA, acetylcholine provocation, thermodilution assessment with the CoroFlow Cardiovascular System, and intravascular imaging. This is one of the only studies of patients with INOCA that includes intravascular imaging with IVUS and OCT to assess for myocardial bridging, quantify atherosclerotic plaque burden, and characterize plaque morphology to determine whether cardiovascular events are related to the physiology of an underlying vasomotor disorder or the natural history of atherosclerotic cardiovascular disease.

Prior studies have demonstrated a wide range for the incidence of adverse cardiovascular events in patients with INOCA^7^; however, the hazard of so-called “hard events” has generally been reported to be lower than that of patients with obstructive CAD.^8^^,^^14^ This contrasts with the high burden of symptoms and repeat cardiovascular testing seen in this population.^4^ In DISCOVER INOCA, short-term and long-term outcomes will be assessed for change in response from baseline for patient-reported outcome measures including Seattle Angina Questionnaire, EuroQol 5 Dimensions – 5 Levels, Patient Health Questionnaire-8, and Generalized Anxiety Disorder-7, as well as for procedural complications, progression of CAD, changes in medication over time, and MACE. The data elements included in this study will help characterize the natural history and prognosis of patients with INOCA with patient-reported outcomes such as angina burden and quality of life, as well as with adverse cardiovascular events (including re-presentation for chest pain or invasive angiography). A centralized call center was developed to ensure that patient follow-up includes symptom assessment and serial assessment of patient-reported outcome measures and to capture hospitalizations or cardiac procedures regardless of the location of care.

DISCOVER INOCA is complimentary to other active registry studies of patients with INOCA. The Inclusive Invasive Physiological Assessment in Angina Syndromes Registry (ILIAS, NCT04485234) is a global registry with enrolling sites in Korea, the Netherlands, Japan, Spain, Denmark, Italy, and the United States.^15^^,^^16^ Participants in ILIAS underwent CFT according to the local protocol of the enrolling institution, data was entered into a shared electronic data capture system, and patient follow-up was performed by the enrolling site. More than 2000 patients with stable ischemic heart disease or acute coronary syndromes (nonculprit vessels only) who underwent assessment of coronary pressure and flow in at least 1 vessel have been enrolled. In contrast to ILIAS, DISCOVER INOCA will exclude patients with myocardial infarction and has a prespecified diagnostic protocol. The Ischemia in Patients With Nonobstructive Disease (INOCA) in Italy INOCA IT Multicenter Registry (INOCAIT, NCT05164640) is a prospective study of 200 consecutive patients with INOCA at 3 centers in Italy.^17^ All subjects in INOCAIT will undergo assessment of CFR and IMR followed by provocative testing with acetylcholine. The Coronary Microvascular Disease Registry (NCT05960474) is a retrospective/prospective descriptive study of patients with stable ischemic heart disease and acute coronary syndromes who undergo invasive physiologic assessment of the microvasculature.^18^

These efforts demonstrate the intensive global efforts to characterize INOCA, which is a prevalent and increasingly recognized syndrome that has historically been poorly defined and challenging to treat. To date, 100 participants have been enrolled in DISCOVER INOCA, and the dissemination of results is anticipated in early 2025. The completion of this study will add to the growing body of evidence defining the patient population, physiologic phenotypes, and long-term prognosis of INOCA.
