# Supplementary material for: Determining the Cause of Coronary Vasomotor Disorders in Patients With Ischemia and Nonobstructive Coronary Arteries: Design and Rationale of the DISCOVER INOCA Prospective, Multicenter Registry
Source: J Soc Cardiovasc Angiogr Interv. 2024 May 3;3(6):102046. doi: 10.1016/j.jscai.2024.102046 (PMC11308755; doi:10.1016/j.jscai.2024.102046)
Supplement: Supplemental Data [file mmc1.docx]

Supplemental Materials

**Supplemental Table S1. Enrolling Sites**

|  | **Site Name** | **Site Investigator** |
| --- | --- | --- |
| 1 | Yale University, Connecticut | Samit Shah, MD |
| 2 | The Christ Hospital, Ohio | Timothy Henry, MD |
| 3 | Thomas Jefferson University Hospital, Pennsylvania | Michael Savage, MD |
| 4 | Columbia University Irving Medical Center, New York | Megha Prasad, MD |
| 5 | NYU Langone Health, New York | Nathaniel Smilowitz, MD |
| 6 | New York-Presbyterian – Brooklyn Methodist Hospital, New York | Yuhei Kobayashi, MD |
| 7 | Northeast Georgia Medical Center, Georgia | Habib Samady, MD & Glen Henry, MD |
| 8 | Stanford University Medical Center, California | Jennifer Tremmel, MD |

**Supplemental Table S2. Study Schedule of Procedures and Assessments**

|  | **Screening / Baseline** | **Index Procedure**  **(day 0)** | **Post-procedure (within 48 hours)** | **30 days**  **(30 ± 7 days)** | **6 months**  **(± 30 days)** | **12 months**  **(± 30 days)** | **2, 3, 4, 5 years**  **(± 60 days)** |
| --- | --- | --- | --- | --- | --- | --- | --- |
|  | Clinic  Visit | Clinic  Visit | Clinic  Visit | Telephone Contact  ± Clinic Visit | Telephone Contact  ± Clinic Visit | Telephone Contact ± Clinic  Visit | Telephone Contact  ± Clinic Visit |
| **General eligibility criteria** | ● |  |  |  |  |  |  |
| **Informed consent** | ● |  |  |  |  |  |  |
| **Medical history** | ● |  |  |  |  |  |  |
| **Concomitant medications** | ● | ● | ● | ● | ● | ● | ● |
| **12-lead ECG^1^** | ● |  |  |  |  |  |  |
| **Laboratory testing^2^** | ● |  |  |  |  |  |  |
| **Coronary angiography^3^** |  | ● |  |  |  |  |  |
| **Angiographic eligibility criteria^3^** |  | ● |  |  |  |  |  |
| **Physiology Assessment and IVUS/OCT^4^** |  | ● |  |  |  |  |  |
| **Adverse events** |  | ● | ● | ● | ● | ● | ● |
| **Angina assessment (CCS, SAQ)** |  | ● |  | ● | ● | ● | ● |
| **EQ-5D-5L** |  | ● |  | ● | ● | ● | ● |
| **PHQ-8** |  | ● |  | ● | ● | ● | ● |
| **GAD-7** |  | ● |  | ● | ● | ● | ● |

^1^ The most recent screening/baseline ECG prior to procedure will be recorded

^2^ Pre-procedure laboratory testing should be performed per the facility standard of care, which may include cardiac biomarkers, CBC, serum chemistry, lipid panel, cardiac biomarkers, and coronavirus 2019 (COVID-19) testing

^3^ All angiographic films of the index procedure must be forwarded to the Angiographic Core Laboratory. If repeat coronary angiography is performed for clinical indications at any time during follow-up, it is requested that angiographic films (and any intracoronary imaging) are also submitted.

^4^ All index procedure intracoronary imaging (including IVUS and OCT) and physiology measurements must be forwarded to the Imaging Core Laboratory. If repeat coronary angiography is performed for clinical indications at any time during follow-up, it is requested that these subjects undergo repeat physiologic assessment and intracoronary imaging with the same modalities used for the initial procedure, and that these data are also submitted.

## Supplemental Appendix

## Index Procedure

### Angiographic Visualization

Diagnostic angiography, including choice of vascular access, should be performed per the facility standard of care. Angiographic imaging will follow the standardized acquisition protocol:

- Coronary angiograms should include at least two views (orthogonal or at least 30 degrees apart) of each major epicardial coronary vessel. The severity of stenosis in the vessel at the time of the procedure will be determined by the treating physician and reported in the cardiac catheterization report.
- Subjects who are found to have an angiographic stenosis ≥ 70% in a major epicardial vessel by visual estimation will be excluded based on pre-specified exclusion criteria and no further data will be collected.
- If a myocardial bridge is visualized this should be reported.

If access is obtained in the radial artery a vasodilator cocktail (including nitroglycerin, nitroprusside, or calcium channel blockers) should not be administered prophylactically. If there is radial artery spasm that prohibits catheter advancement, intra-arterial nitroglycerin alone may be administered and coronary thermodilution should be performed first followed by provocative testing with acetylcholine a minimum of 10 minutes after nitroglycerin administration. Administration of a radial vasodilator cocktail, including the agents and doses, should be reported. Anti-coagulation should be administered per the cardiac catheterization laboratory standard of care.

- Vessel selection for physiologic evaluation and intracoronary imaging should be determined based on clinical indications, such as ischemia in a vascular territory on a non-invasive test or localizing electrocardiographic changes.
- If a patient has an epicardial stenosis that would warrant further physiologic assessment with FFR or RFR by standard of care indications, that vessel should undergo further evaluation for inclusion into the registry.
- For angiographic stenoses ≥ 50% but < 70%, FFR and RFR must be performed. If the FFR is ≤ 0.80 or the RFR is ≤ 0.89 the subject will be excluded based on pre-specified exclusion criteria and no further data will be collected.
- If the coronary arteries appear angiographically normal and there is no clinical indication for evaluating a specific vessel, then the left anterior descending (LAD) artery is the preferred vessel for guidewire testing and intracoronary imaging
- At least one vessel should be interrogated with physiology testing and intracoronary imaging.
- Evaluation of additional vessels is at the discretion of the operator and all additional angiographic, physiologic assessment, and intracoronary imaging should be transferred to the core laboratory as per the initial vessel.

### Acetylcholine Testing

- In the majority of cases provocative testing with acetylcholine (ACh) should be performed after diagnostic angiography and before guidewire-based physiology or intracoronary imaging. However, the sequence of testing will be based on operator preference and should be documented according to the angiographic acquisition sequence labeling guidelines.
- Provocative testing should preferentially be performed on the left coronary artery due to an increased risk of bradycardia and tachyarrhythmias with provocative testing of the right coronary artery.
- Patients should be monitored with electrocardiographic monitoring according to the catheterization laboratory routine. Additional monitoring with a cardiac monitor with pacing / defibrillator functionality is recommended.
- ACh should be administered by a gradual bolus hand injection via a 6Fr guide catheter or micro-infusion pump and patients should be monitored for bradycardia, atrioventricular block, tachyarrhythmias, or symptoms such as chest pain.
- After a minimum of 30 seconds the patient should be assessed for any new symptoms and an electrocardiogram should be obtained from the telemetry monitor (ideally 12-lead if possible).
- After each dose a cine angiogram should be performed in the exact same projection as the baseline angiogram. The angiographic view is per the discretion of the operator.
- After waiting for at least one minute the dose should be escalated to the next appropriate dose, and a minimum of two doses are recommended for each patient.
- If the patient experiences chest discomfort, there is > 1mm ST-elevation or > 1mm ST-depression on the EKG, and there is ≥ 90% vasospasm of the epicardial vessel then the test is diagnostic for VSA and no further doses should be performed.
- Patients may experience ≥ 90% narrowing of the epicardial vessel in the absence of symptoms or ischemic EKG changes and this is also consistent with VSA and additional doses are per the discretion of the operator
- Microvascular spasm includes angina and/or ischemic EKG changes in the absence of spasm of the epicardial vessel during acetylcholine infusion

Recommended ACh doses are as follows:

**Table:** Acetylcholine Dosing

| **Bolus Injection** | Initial Dose | Second Dose | Maximum Dose* |
| --- | --- | --- | --- |
| **Left coronary artery** | 20µg | 100µg | 200µg |
| **Right coronary artery** | 2µg | 20µg | 50µg |
| **Micro-infusion Pump** | 10^–6^ mol/l | 10^–5^ mol/l | 10^–4^ mol/l** |

*Of note, women are less likely than men to have a dose-dependent response to ACh doses > 100µg, but if diagnostic criteria have not been met, then a maximum of 200µg should be administered to subjects of either sex [28].

**If graded doses are performed using a micro-infusion pump and there is no evidence of epicardial or microvascular spasm at the highest infused dose, a bolus 200µg acetylcholine dose should be administered via the guide or microcatheter

- The initial dose (i.e., 20µg or 2µg) should be administered slowly (over 60 seconds) and the patient monitored for arrhythmia or hemodynamic changes. The subsequent doses could be administered at a higher rate (over 30 seconds) at the discretion of the operator.
- If the patient develops transient sinus pauses, heart block, atrial tachyarrhythmia, or hypotension during ACh administration the rate of infusion should be slowed, but not discontinued. If the dose is aborted due to arrhythmia, hemodynamic intolerance, or patient symptoms the dose and adverse effects should be recorded.
- Use of a temporary pacemaker during provocative testing is at the discretion of the operator. If a temporary pacemaker is inserted either in anticipation of bradyarrhythmia or as a result of bradyarrhythmia this should be reported.
- After the highest administered dose of ACh is complete the capacity for endothelium-independent vasodilatation should be assessed with a bolus injection of 200µg of intracoronary nitroglycerin. After waiting approximately 60 seconds for a hemodynamic effect (such as a decrease in blood pressure) a repeat cine angiogram should be performed in the same views used for ACh testing. If there is persistent epicardial vasospasm or slow flow then additional doses of nitroglycerin should be administered until spasm is relieved, and the additional doses should be reported.

### Coronary Thermodilution

A 6Fr guide catheter should be used for coronary thermodilution and guidewire-based assessment of FFR, RFR, CFR, and IMR. The guide should be coaxial with the vessel of interest and the pressure waveform should be evaluated to avoid pressure dampening. Caution is advised if there is an ostial stenosis as there may be subtle dampening of the pressure.

- Prior to performing guidewire-based assessment at least 200 µg of intracoronary nitroglycerin must be administered to correct for any wire-induced spasm and flow-mediated vasodilation that may be induced during adenosine infusion.
- If intracoronary nitroglycerin was administered after ACh testing then no further dosing is required.

Therapeutic anticoagulation should be maintained according to the facility standard of care. A PressureWire-X should be prepared per the Instructions for Use (IFU) and advanced 2 cm into the proximal vessel so the proximal aspect of the radio-opaque tip is just outside of the ostium of the guide catheter.

- If a needle introducer is used it should be removed and the guide should be flushed with 10cc of saline prior to equalization of the pressure tracings.
- After equalization the wire should be advanced into the distal vessel as far as safely possible (such that the proximal aspect of the radio-opaque segment is a minimum of 6 cm distal to the guide tip and the tip of the wire is at least ~9 cm from the guide tip). If the wire cannot be advanced due to vessel tortuosity another vessel should be evaluated.

The guide should then be flushed with 10 cc of room temperature saline as a “cool down” to remove blood and contrast media from the guide and the temperature should be zeroed. The resting heart rate, Pd, Pa, and RFR should be recorded.

- After the cool down thermodilution should be performed by injecting 3 mL of room temperature saline into the coronary artery with a 3 mL Luer lock syringe.
- Injections should be smooth and brisk. If the thermodilution curve appears appropriate perform 2 additional injections.
- The average of three injections is the thermodilution mean transit time (Tmn). There should be less than 15% variance between measurements and if there is significant variation injections can be repeated.

Induce maximal hyperemia with intravenous adenosine at 140 µg/kg/min administered via an antecubital or other large peripheral vein (at least 20g peripheral intravenous catheter) or central venous access if available.

- Perform a second “cool down” with 10 cc of room temperature saline and repeat the thermodilution procedure as described. Three (3) additional injections should be performed to determine the hyperemic mean transit time (Tmn-hyp).
- The ratio of the Tmn to the Tmn-hyp is the CFR, and the product of the Tmn-hyp and the mean distal coronary pressure Pd during hyperemia is the IMR. The FFR will be calculated as the ratio of the P_d_ divided by the P_a_ during hyperemia.
- After thermodilution is completed stop the adenosine infusion and record the total duration of adenosine administered.

After intracoronary imaging is completed an RFR pullback should be performed to evaluate for the presence of diffuse physiologically significant disease. If imaging is performed over a workhorse coronary guidewire rather than the PressureWire X the RFR pullback should performed when the PressureWire X is removed. To evaluate for the presence of drift the proximal aspect of the radio-opaque segment of the wire should be positioned just outside of the guide tip and the Pd/Pa should be recorded.

### Intracoronary Imaging

Intracoronary imaging should be performed on the vessel that underwent guidewire-based physiology assessment according to the acquisition guidelines. Where available OCT is the preferred imaging modality.

- In cases where OCT is not available or OCT is suboptimal for clinical reasons IVUS may be performed. If IVUS is performed imaging should be obtained with an automated pullback at 0.5mm/sec and with a minimum of 45 mHz imaging transducer.
- OCT may not be suitable for patients with significant renal dysfunction or chronic kidney disease due to the requirement for contrast media administration
- OCT may not be possible in vessels with severe tortuosity, in which case IVUS should be performed preferentially
- OCT may not be optimal in vessels with a large diameter > 5mm due to challenges with clearing blood with contrast media, and IVUS should be performed preferentially

For OCT the target vessel should be a minimum of 2 mm in diameter. After confirming that the vessel can be adequately cleared of blood with contrast media imaging should be performed by automated pullback (36 mm/s for “survey” mode with 0.2mm slice thickness) during injection of contrast media.

For IVUS or OCT the imaging catheter should be positioned to image any segments where vasospasm is present during provocative testing with ACh. If there was no significant vasospastic response to ACh then the catheter should be positioned as distally as safely possible in the target vessel.

- OCT images acquired with the newly released Ultreon software are **not** suitable for offline analysis and cannot be used for this study
- A minimum of 54mm should be imaged, and preferentially, > 70mm lengths of imaging should be performed if clinically safe and feasible
- If a myocardial bridge is present then imaging should include the intramyocardial segment and segments proximal/distal to the bridge
